# Prevalence and clinical impact of mono- and co-infections with endemic coronaviruses 229E, OC43, NL63, and HKU-1 during the COVID-19 pandemic

**DOI:** 10.1016/j.heliyon.2024.e29258

**Published:** 2024-04-07

**Authors:** I. Trifonova, N. Korsun, I. Madzharova, P. Velikov, I. Alexsiev, L. Grigorova, S. Voleva, R. Yordanova, I. Ivanov, T. Tcherveniakova, I. Christova

**Affiliations:** a National Laboratory “Influenza and ARD”, Department of Virology, National Center of Infectious and Parasitic Diseases (NCIPD), Sofia, Bulgaria; b Infectious Disease Hospital “Prof. Ivan Kirov”, Department for Infectious Diseases, Parasitology and Tropical Medicine, Medical University of Sofia, Bulgaria

**Keywords:** Endemic coronaviruses, SARS-CoV-2, Respiratory infections, Co-infections

## Abstract

**Introduction:**

Endemic human coronaviruses (eHCoVs) are found worldwide and usually result in mild to moderate upper respiratory tract infections. They can lead to more severe illnesses such as croup, bronchiolitis, and pneumonia in vulnerable populations. During the coronavirus disease 2019 (COVID-19) pandemic, information on HCoV prevalence and incidence and clinical impact of co-infections of HCoV with SARS-CoV-2 was lacking.

**Objectives:**

Thus, this study aimed to determine the prevalence and clinical significance of infections caused by eHCoVs during the COVID-19 pandemic in Bulgaria.

**Methods:**

From January 2021 to December 2022, nasopharyngeal swabs of patients with acute upper or lower respiratory tract infections were tested for 17 respiratory viruses using multiplex real-time polymerase chain reaction assays. The clinical data and laboratory parameters of patients infected with respiratory viruses were analysed.

**Results:**

Of the 1375 patients screened, 24 (1.7 %) were positive for HCoVs, and 197 (14.3 %) were positive for eight other seasonal respiratory viruses. Five (0.7 %) of 740 patients positive for SARS-CoV-2 were co-infected with eHCoVs. Co-infected patients had a mean C-reactive protein level of 198.5 ± 2.12 mg/mL and a mean oxygen saturation of 82 ± 2.8 mmHg, while those in patients co-infected with SARS-CoV-2 and other respiratory viruses were 61.8 mg/mL and 92.8 ± 4.6 mmHg, respectively (*p* < 0.05). Pneumonia was diagnosed in 63.3 % of patients with HCoV infection and 6 % of patients positive for other seasonal respiratory viruses (*p* < 0.05). Patients with SARS-CoV-2 mono-infection stayed in hospital for an average of 5.8 ± 3.7 days, whereas the average hospital stay of patients with eHCoV and SARS-CoV-2 co-infection was 9 ± 1.4 days (*p* < 0.05).

**Conclusion:**

These findings indicate the low prevalence of eHCoVs and low co-infection rate between eHCoVs and SARS-CoV-2 during the COVID-19 pandemic in Bulgaria. Despite their low incidence, such mixed infections can cause severe signs that require oxygen therapy and longer hospital stays, underlining the need for targeted testing of severe COVID-19 cases to identify potential co-infections.

## Highlights

• Low prevalence of endemic HCoVs during the COVID-19 pandemic.
• Low rate of HCoV co-infections in patients positive for SARS-CoV-2.
• Hospitalised patients with eHCoV exhibit symptoms similar to those with SARS-CoV-2.
• Severe COVID-19 has been observed in co-infected hospitalised patients with HCoV.

## 1 Introduction

Human coronaviruses (HCoVs) cause respiratory diseases of varying severities. To date, seven CoV types are known to infect humans: four endemic, low pathogenic HCoVs, 229E, NL63, OC43, and HKU-1, and three highly pathogenic HCoVs, severe acute respiratory syndrome coronavirus (SARS-CoV-1), Middle East respiratory syndrome coronavirus (MERS-CoV), and SARS-CoV-2. Endemic HCoVs (eHCoVs) are globally distributed and usually cause mild to moderate self-limiting upper respiratory tract infections (URTI), known as the common cold [1]. More severe illnesses, including croup, bronchiolitis, and pneumonia, have been reported in infants, young children, the elderly, and immunocompromised individuals [2]. The first HCoVs, 229E and OC43, were identified in the 1960s [3], and NL63 and HKU-1 were discovered in the Netherlands in 2004 [4] and Hong Kong in 2005 [5], respectively. SARS-CoV-1, responsible for the 2002/2003 epidemic, emerged in November 2002 in China and caused 8096 cases of the disease in 27 countries, including 774 deaths (mortality rate, 9.6 %) [6]. MERS-CoV emerged in the Arabian Peninsula in September 2012 and was associated with the highest fatality rate (36 %) compared to the coronaviruses known to date [7]. SARS-CoV-2 emerged in Wuhan, China, in December 2019 and caused the largest pandemic in the last 100 years, resulting in multitudinous human casualties and serious economic disruptions [8].

**Fig. 1:**
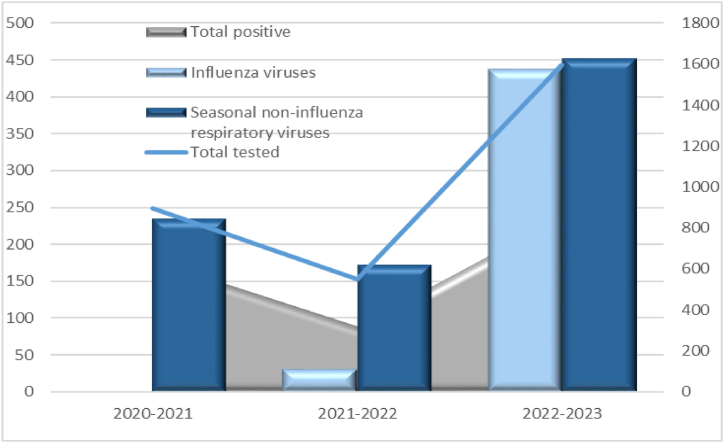
Distribution of proven influenza and non-influenza respiratory viruses during the period 2020–2021, 2021–2022 and 2022–2023 in Bulgaria.

Various nonpharmaceutical measures implemented to reduce SARS-CoV-2 transmission resulted in a significant decline in the spread of seasonal respiratory viruses [[9], [10], [11]]. Globally, the incidence of influenza viruses drastically reduced during the winter seasons of 2020/2021 and 2021/2022 [[12], [13], [14], [15]]. Fig. 1 depicts the decline in the incidence of influenza viruses in Bulgaria during this period [15].

Epidemiologic studies conducted before the coronavirus disease 2019 (COVID-19) pandemic have shown that eHCoVs are detected in 5–30 % of patients with URTI [16,17]. During the pandemic, the prevalence of the four common HCoVs was <2 % [18]. Often, eHCoVs are found co-infecting with other respiratory pathogens. Previous studies have reported a co-detection rate of 30–50 % among patients positive for HCoVs [19,20]. The weak circulation of these viruses during the COVID-19 pandemic resulted in a reduced number of co-infections, including that of SARS-CoV-2 [21,22]. In a previous study conducted by us, it was reported that individuals who were infected with both SARS-CoV-2 and another respiratory virus had a more severe clinical course and required more frequent hospitalisation or invasive treatment [23]. Other studies have also reported a more severe clinical picture in patients with COVID-19 who were co-infected [[24], [25], [26]]. Based on laboratory findings, imaging studies, and disease outcomes, some authors showed that co-infections do not aggravate the condition of the patient [27].

During the COVID-19 pandemic, information on the prevalence of the common HCoVs and the frequency and clinical impact of SARS-CoV-2 co-infections was limited. Therefore, uniquely in our study, we aimed to determine the incidence of mono- and co-infections with eHCoVs, analysed and compared the clinical data of patients with mono infections and those co-infected with SARS-CoV-2 and to evaluate the clinical impact of co-infections with HCoVs and SARS-CoV-2.

## 2 Materials and methods

### 2.1 Study design

This study was conducted from January 2021 to December 2022 in Bulgaria to determine the prevalence and clinical advice of eHCoV during the COVID-19 pandemic. Patients (inpatient and outpatient) from all age groups and different regions of Bulgaria were included in the study and were classified based on their SARS-CoV-2 infection status. Data about demographics, clinical attributes, treatment regimens, and laboratory outcomes were extracted from the electronic medical records. The study protocol was approved by the institutional ethics committee (National Center of Infectious and Parasitic Diseases Institutional Review Board/Institutional Ethics Commit Number IRB 00006384 protocol number 5/2022), and each participant or their guardians provided informed consent.

### 2.2 Population and sampling

Samples were taken from patients with respiratory symptoms via nasopharyngeal swabs. Clinical and epidemiological data were prospectively collected from both hospitalised and outpatient patients from different regions of the country. A 2 mL media swab (polyester collection swabs) was used to collect the sample. Samples were transported on ice and stored at 4 °C for up to 72 h before shipping to the National Laboratory “Influenza and ARI”. Samples were processed immediately or, if not possible, stored at −80 °C until further testing.

### 2.3 Exclusion criteria

Exclusion criteria were established for this study. Patients who were unwilling or unable to provide consent or those unable to undergo a nasopharyngeal sampling were excluded from the study. Additionally, any samples that did not meet the transportation and storage criteria for nasopharyngeal samples outlined in the study were excluded upon receipt in the laboratory.

### 2.4 Clinical data and definitions used

Assessment of the patient's condition was made according to a category scale: 1 –not hospitalised with the resumption of normal activity; 2 - not hospitalised, but cannot resume normal activities; 3 - hospitalised, without need for additional oxygen; 4 - hospitalised, requiring additional oxygen; 5 - hospitalised requiring high-flow nasal oxygen therapy, non-invasive mechanical ventilation, or both; 6 - hospitalised requiring extracorporeal membrane oxygenation, invasive mechanical ventilation, or both; 7 – death [28].

The epicrisis of patients in hospital facilities was analysed by specialists in the field of respiratory medicine. The diagnosis of pneumonia was based on radiographic examination and the presence of pulmonary infiltrates, and the definition of acute respiratory distress was based on the Berlin definition [29].

Outpatient data were collected in questionnaire formats, which contained data including age, gender, and clinical manifestations. The clinical manifestations studied included fever, cough, malaise, sore throat, chest pain, rhinorrhoea, convulsions, headache, and abdominal discomfort.

### 2.5 Nucleic acid extraction and real-time polymerase chain reaction (PCR)

Viral DNA and RNA were extracted by an automated extraction system using the ExiPrep Dx Viral DNA/RNA kit (Bioneer, Daejeon, Republic of Korea) SaMag 12 System (Sacace Biotechnologies, Italy).

The respiratory viruses were detected using two real-time RT-PCR devices: the QuantStudio™ 3 Real-Time PCR System, 96 Wells (ThermoFisher Scientific), and the CFX96 Touch Real-Time PCR Detection System (Bio-Rad). To capture four eHCoVs, we used a multiplex mask containing SuperScript III Platinum ® One-Step qRT-PCR System (Invitrogen, USA) and prime and probes 229E TexasRed + OC43 Hex + NL63 Fam + HKU-1Cy5.

For SARS-CoV-2 detection, we used two kits, the Multiplex Real-time RT-PCR Kit (FluSC2) for simultaneous detection of influenza A/B and SARS-CoV-2 viruses and the TaqPath COVID-19 CE-IVD PCR Kit (Thermo Fisher Scientific), to identify three regions in the SARS-CoV-2 genome: N-gene, S-gene and the ORF ab.

To detect eight respiratory viruses: respiratory syncytial virus (RSV), human metapneumovirus (HMPV), parainfluenza virus type (PIV) 1/2/3, rhinovirus (RV), adenovirus (AdV), and bocavirus (BoV), we use a tree multiplex PCR mix with combinations that include prime and probe and SuperScript III Platinum ® One-Step qRT-PCR System (Invitrogen, USA).We have described the preparation of the primer and probe mixes in of our earlier articles [30].

Mix 1: AdV + RSV + PIV1

Mix2: BoV + RV + PIV2

Mix 3: HMPV + PIV3

Primers were synthesised based on primer sequences reported earlier [31]. The result of real-time RT-PCR analyses was considered positive at Сt ≤38.

### 2.6 Statistical analysis

Chi-square or Fisher's exact tests were used for statistical analyses and results were reported as total numbers and percentages. Noncontinuous variables were evaluated using Fisher's least significant difference pairwise comparison. Statistical significance was set at *p* < 0.05. All analyses were performed using the Origin Data Analysis and Graphing statistical software package.

## 3 Results

### 3.1 Patient characteristics

A total of 1375 patients with ARI (673 males and 702 females; median age, 36 years; range: 45 days to 98 years) were enrolled in this study. Of these, 213 (15.5 %) were outpatients and 1162 (84.5 %) were inpatients. The patients were categorised into two groups based on their SARS-CoV-2 test results: 740 positive and 635 negative for SARS-CoV-2. The patients were divided into four age groups: 0–5 years old (356, 25.9 %), 6–16 years old (258, 18.8 %), 17–64 years old (377, 27.4 %), and ≥65 years old (384, 27.9 %). Of the study participants, 49 % were male and 51 % female.

### 3.2 Distribution of endemic human coronaviruses

Of the 1375 patients examined, 24 (1.7 %) were positive for eHCoVs and 197 (14.3 %) were positive for the other eight seasonal respiratory viruses. The number of patients infected with NL63, OC43, 229E, and HKU-1 was 11 (0.8 %), 8 (0.6 %), 3 (0.2 %), and 2 (0.1 %), respectively. The positive rate of HCoVs among hospitalised patients was higher than that among outpatients (1.9 % vs. 0.9 %) (*p* < 0.05).

### 3.3 Age and weekly distribution of endemic human coronavirus infections

**Fig. 2:**
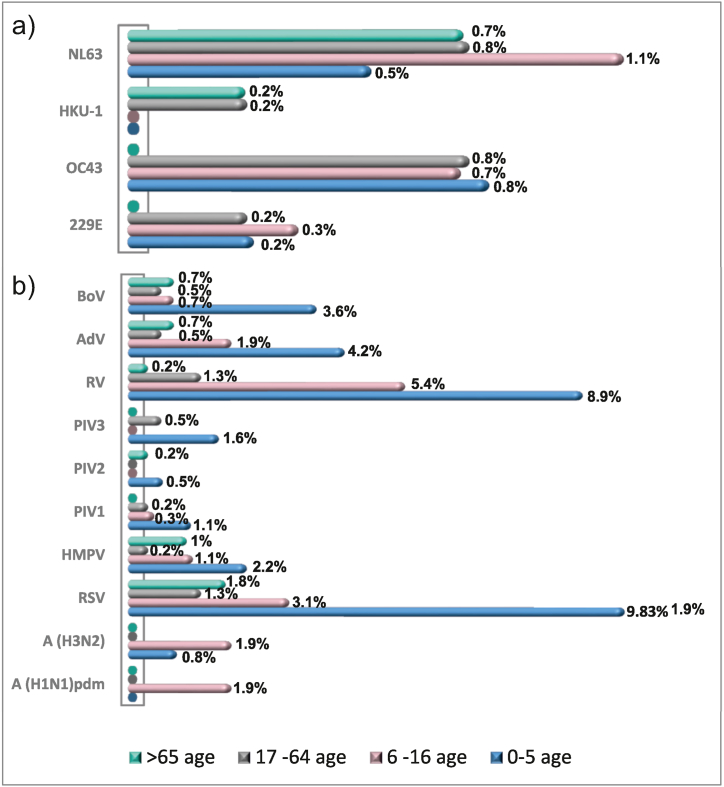
The percentage distribution of patients in five age groups: 0–5, 6–16, 17–64, and >65 years, based on the proportion infected with two virus categories in 2021/2022. The first category included a) endemic HCoVs such as NL63, HKU-1, 229E, and OC43, while the second category b) included other seasonal respiratory viruses such as BoV, AdV, RV, PIV1,2,3, HMPV, RSV, A (H3N2), and A (H1N1)pdm.

At least one of the four eHCoVs was identified in each age group, and only NL63 was detected in all the age groups (Fig. 2a). The positivity rate for all four HCoVs was highest in the 6–16-year-old age group (2.3 %; 6/258). The median age of the HCoV-positive cases was 22.5 years (2 days–75 years). No significant difference was observed between male and female HCoV detection rates. Children aged 0–5 years were most affected by other seasonal non-influenza respiratory viruses, accounting for 33.1 % (118/356) of all studied cases (Fig. 2b).

**Fig. 3:**
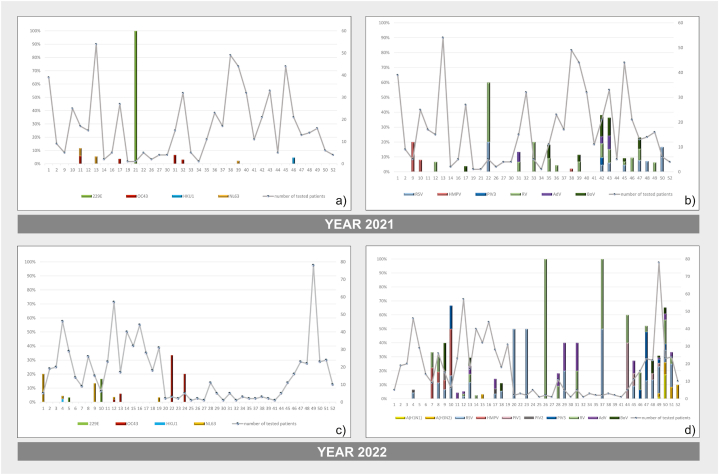
Dynamics in the distribution of endemic HCoV and other seasonal respiratory viruses during the 2021/2022 period. The percentage distribution by a week of those infected with the following viruses a) NL63, HKU-1, 229E, and OC43 in 2021; b) BoV, AdV, RV, PIV1,2,3, HMPV, RSV, A (H3N2) and A (H1N1) in 2021; c)NL63, HKU-1, 229E and OC43 in 2022; d) BoV, AdV, RV, PIV1,2,3, HMPV, RSV, A (H3N2) and A (H1N1) in 2022.

In 2021, the incidence of endemic eHCoVs was higher in the spring, from mid-March to May, with the highest incidence occurring in May (Fig. 3a). Seasonal respiratory infections were more common in autumn (Fig. 3b–d), whereas eHCoVs remained undetected or had a low incidence. In 2022, two spikes were observed in the prevalence of eHCoVs: in March (weeks 9–10) and June (weeks 22–24) (Fig. 3c).

### 3.4 Co-infections involving endemic human coronaviruses

Of the 1375 patients examined, 72 (5.2 %) cases of co-infections were detected. Among 740 patients positive for SARS-CoV-2, 50 (6.8 %) had an additional respiratory viral infection. Five (10 %) of these co-infections involved eHCoVs.

Of the 635 patients negative for SARS-CoV-2, 22 (3.5 %) were co-infected with two or three seasonal respiratory viruses. Four (18.2 %) non-SARS-CoV-2 mixed infections were associated with HCoV infections (Table 1). The co-detected non-coronaviruses were RSV, PIV3, and RV + AdV in 2, 1, and 1 cases, respectively.

**Table 1:** Distribution of patients with mono and co-infections with eHCoVs within the four age groups.

Age groups	Monoinfections; n (%)				Co-infections with SARS-CoV-2; n (%) (n = 5)				Co-infections with other respiratory viruses; n (%)			
(years)	(n = 15)								(n = 4)			
	229Е	OC43	HKU-1	NL63	229Е	OC43	HKU-1	NL63	229Е	OC43	HKU-1	NL63
0–5	1 (6,7)	1 (6.7)		1 (6.7)						2 (50)		1 (25)
0–16	1 (6.7)	1 (6.7)		2 (13.3)		1 (20)		1 (20)				
17–64		2 (13.3)	1 (6.7)	3 (20)		1 (20)			1 (25)			
>65				2 (13.3)			1 (20)	1 (20)				

This study identified at least one of the 4 coronaviruses in each age group (0–5, 6–16, 17–64, and 65+ years). Of the 4 endemic coronaviruses, only NL63 infection was demonstrated in patients of all age groups, with more frequent such infection occurring in children and adolescents aged 6–16 years (1.16 %). The share of the 4 coronaviruses is highest among patients aged 6–16 years – (2.33 %), followed by that of adolescents and patients of active age (17–64) – (2.12 %). In the youngest children and patients over 65 years of age, the proportion of evidence of such infections was relatively low: 0–5 (1.69 %) and >65 (1.04 %). Regarding the variety of established coronavirus infections and in patients over 65 years of age, only two endemic coronavirus pathogens have been proven: HCoV-HKU-1 (0.26 %) and HCoV-NL63 (0.56 %).

**Fig. 4:**
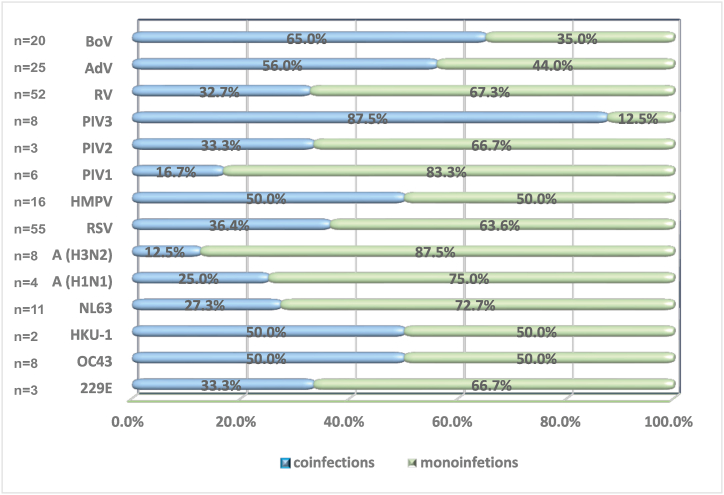
Distribution of the detected seasonal respiratory viruses according to their involvement in mono- and co-infections.

In contrast to the map of the spread of epidemic coronaviruses, children aged 0 to 5 had the highest share of infections caused by other respiratory viruses - 33.15 %. In patients aged 17–64 years, evidence of non-coronavirus respiratory infection was lowest (4.77 %) (Fig. 4).

Regarding the distribution of proven co-infections and mono-infections, it can be seen from Table 1 that in children aged 0–5 years, no mixed infections with SARS-CoV-2 have been proven, while mixed infections in combination with еHCoV, etc. respiratory viruses were proven in this age group in 75 % of cases of such infection (p = 0.04). In adults over 65 years of age, only 2 (40 %) of mixed infections with the participation of SARS-CoV-2 were proven, while those with the study of other non-coronavirus respiratory infections were not proven (p = 0.4444).

### 3.5 Clinical manifestations and laboratory data of patients infected with respiratory viruses

Clinical and laboratory data were collected from electronic medical records of 432 and 364 patients, negative and positive, respectively, for SARS-CoV-2. Clinical information was available for only 14 (28 %) patients co-infected with SARS-CoV-2 and other respiratory viruses and two (40 %) patients with SARS-CoV-2 and eHCoV co-infections.

Clinical manifestations of respiratory diseases, such as fever, cough, rhinitis, and dyspnoea, were observed in a greater percentage of patients positive for SARS-CoV-2 than in those with detected non-CoV respiratory infections (*p* < 0.05). Patients with SARS-CoV-2 co-infections also had a higher incidence of these symptoms, including headaches, than patients with non-CoV respiratory infections (*p* < 0.05). Dyspnoea was also reported more often among patients positive for SARS-CoV-2 and eHCoVs than among patients with non-CoVs (*p* < 0.05). Among patients diagnosed with pneumonia, SARS-CoV-2 and eHCoVs were identified more frequently than other respiratory viruses (*p* < 0.05) (Table 2).

**Table 2:** Comparison of clinical manifestations, diagnosis, and associated complications observed in patients with detected respiratory viruses.

	SARS-CoV-2 mono infections	Co-infections SARS-CoV-2 with non-CoV respiratory virus	Endemic HCoVs	Non-CoV respiratory viruses	P-value SARS-CoV-2 mono vs. SARS-CoV-2 co-infections	P-value SARS-CoV-2 mono infections vs. non-CoV respiratory viruses	P-value SARS-CoV-2 co-infections vs. non-CoV respiratory viruses	P-value Non-CoV respiratory viruses vs. endemic HCoVs
	(n = 350)	(n = 14)	(n = 19)	(n = 100)				
	(n, %)	(n, %)	(n, %)	(n, %)				
**Clinical manifestations**								
Fever	276 (78)	13 (92)	7 (36)	38 (38)	0,3167	0,0001	0,0001	1
Cough	263 (75)	10 (71,4)	10 (52,6)	34 (34)	0,7560	0,0001	0,0161	0,1937
Rhinitis	104 (29,7)	5 (35,7)	5 (26,3)	13 (13)	0,7842	0,0007	0,0448	0,1629
Headache	29 (8,2)	4 (28,5)	4 (21)	3 (3)	0,0293	0,0786	0,0042	0,0123
Vomiting	58 (16,5)	3 (21,4)	3 (15,8)	11 (11)	0,7126	0,2086	0,1343	0,6962
Diarrhea	46 (13,1)	3 (21,4)	3 (15,8)	19 (19)	0,4148	0,8802	0,7319	1
Dyspnea	72 (20,5)	5 (35,7)	5 (26,3)	5 (5)	0,71	0,0001	0,0024	0,0093
**Diagnosis and complications of respiratory infections**								
Laryngotracheitis	12 (3,4)	0 (0)	0 (0)	7 (7)	1	0,1539	0,5944	0,5957
Bronchitis	52 (14,8)	0 (0)	0 (0)	3 (3)	0,2347	0,0008	1	1
Bronchiolitis	0 (0)	0 (0)	0 (0)	14 (14)	0	0,0001	0,2109	0,1222
Pneumonia	172 (49)	10 (71,4)	12 (63,1)	6 (6)	0,1711	0,0001	0,0001	0,0001

Four (80 %) patients co-infected with SARS-CoV-2 and epidemic HCoV were diagnosed with pneumonia, whereas only two (9 %) of those infected with only eHCoVs had pneumonia (*p* < 0.05). In four (80 %) patients co-infected with eHCoVs, a fever with a body temperature above 38 °C was observed, whereas, in mono-infected patients, the body temperature did not exceed 37 °C (*p* = 0.0001). Moreover, 60 % (9/15) of patients with co-infections had a body temperature above 38 °C (*p* < 0.05). Significant differences were found in the C-reactive protein (CRP) levels and mean saturation between patients positive for SARS-CoV-2, those co-infected with HCoVs, and those co-infected with other respiratory viruses. In the first group of patients, these values were 198.5 ± 2.12 mg/mL and 82 ± 2.8 mmHg, respectively, whereas in the second group, the values were 61.8 ± 79.4 mg/mL and 92.8 ± 4.6 mmHg, respectively (*p* < 0.05). Differences were also found between patients with SARS-CoV-2 mono- and co-infections in the requirement for ventilation support and intensive treatment (*p* < 0.05) (Table 3).

**Table 3:** Laboratory data, treatment, length of stay in the hospital, and clinical adverse events in SARS-CoV-2 mono- and co-infected patients with other respiratory viruses.

	SARS-CoV-2	Co-infections of SARS-CoV-2 and other respiratory viruses (n = 14)	P-value
	mono-infection (n = 350)		
Laboratory data (mean)			
Partial pressure of oxygen, PO2 (mmHg, mean)	91,8	91,3	n.s.^a^
Leukocytes (10^3/μL, mean)	6,5	6,8	n.s.
Lymphocytes (10 ^9/L, mean)	1	0,8	n.s.
C-reactive protein (CRP) (mg/ml, mean)	110	61,8	0,03
**Treatment and clinical course (n, %)**			
Intravenous corticosteroids	112 (32)	5 (35,7)	0,7751
Low-molecular-weight heparin (LMWH)	223 (63,7)	8 (57,1)	0,7781
Antibiotics	224 (64)	11 (78)	0,3943
Pulmonary vasodilators	20 (5,7)	0 (0)	n.s.
Oxygen therapy	154 (44)	7 (50)	0,7855
Mechanical ventilation	6 (1,7)	2 (25)	0,0337
**Length of hospital stay (days, median, IQR)**	5,8	8,2	0,024
**Clinical adverse events**			
Admittance to ICU	6 (1,7)	2 (25)	0,0337
30-day mortality	21 (6)	1 (7,1)	0,5891

a n.s. - non significant.

Patients co-infected with SARS-CoV-2 and eHCoVs tended to stay in the hospital for an average of 9 ± 1.4 days, whereas patients infected with SARS-CoV-2 alone had a shorter hospital stay, averaging 5.8 ± 3.7 days (*p* < 0.05). Unfortunately, 22 (6 %) patients positive for SARS-CoV-2 for whom clinical information was available did not survive, and one (4.5 %) had a co-infection with RSV.

## 4 Discussion

In this study, the epidemiological and clinical characteristics of HCoV infections during the COVID-19 pandemic in Bulgaria were investigated. In addition, the prevalence of HCoV infections and those caused by SARS-CoV-2 or other respiratory viruses were compared. The prevalence of the four eHCoVs found among ARI cases was low (1.7 %). In our previous study, we examined patients over 65 years of age and found that 7.5 % of ARI cases were caused by HCoV [32]. The study also noted that only influenza A and B were detected in a higher proportion of cases, indicating that HCoV is the main cause of ARI in patients over 65 years of age. Certain types of coronaviruses, known as eHCoVs, have been found responsible for a significant proportion of ARIs, causing up to 30 % of all cases of the common cold [33]. The marked decline in HCoV detection is consistent with the overall decline in circulating seasonal respiratory viruses seen in many countries. It can be attributed to the containment measures of COVID-19 [34].

These viruses have a worldwide distribution, their prevalence varies from year to year, with a tendency to be more prevalent during the winter season [35].

The spread of these viruses exhibits no clear seasonality due to the small number of confirmed infections. One of our studies found that the peak months for SARS-CoV-2 in Bulgaria are from December2021/2022 and March2021/2022.

[30]. This peak in SARS-CoV-2 infections coincides with the observation this study reporting an increase in HCoV evidence for March 2021 and March 2022. Another study conducted in Canada also reported an increase in eHCoV infections in the spring of 2021 [11]. Due to the relaxation of restrictions, the incidence of RSV, eHCoVs, and parainfluenza virus infections in many countries returned to pre-pandemic levels in the spring of 2021. As a result, the seasonal peaks were not consistent with what has historically been seen in the winter months. In contrast the circulation of eHCoVs was reduced or absent during periods of intensive public health measures in the winter season, as reported earlier [18].

Herein, NL63 was the most prevalent of the four eHCoV species, followed by OC43 (*p* < 0.05). Surveillance studies conducted in different countries have shown that the distribution of common eHCoV species varies by year and geographical location [20,36,37].

This study also observed a discrepancy between the age distribution of individuals infected with endemic coronaviruses and those infected with other respiratory viruses. In contrast to some previous research reporting a high occurrence of endemic coronavirus infections in children aged 0–5 years [38,39], our current study detected only 1.69 % of such infections. This finding aligns with the behaviour of SARS-CoV-2, known for its relatively low frequency of evidence in the youngest children [40]. In addition, compared to adults, children younger than 5 years of age tend to be more susceptible to viral respiratory infections [41,42].

Herein, HCoV infection was detected in all age groups; however, patients aged 6–16 years had the highest positivity rate (2.3 %). Additionally, similar to another study, the highest proportion of infection in children aged 0–5 and 6–16 years was with OC43 and NL63 [34,43]. The four HCoVs have been shown to frequently co-infect with other respiratory viruses, which was also observed herein. The total co-infection rate was 37.5 % (9/24), concordant with the results of previous studies [19,20,44], even though some authors have reported low co-infection rates during the COVID-19 pandemic [18].

HCoVs have the fifth highest rate of co-infection among viruses studied. All four species are involved in co-infections, but NL63 is identified more frequently. In addition, five (20.8 %) patients with endemic HCoV infections were co-infected with SARS-CoV-2. Pooled data from this study show that respiratory disease symptoms in patients with endemic coronaviruses are more similar to those in patients with proven SARS-CoV-2 than in those infected with other respiratory agents. A similar claim was reported in another study [45]. This study also reported a relatively high rate of pneumonia cases due to epidemic coronaviruses, which are also characteristic of patients infected with SARS-CoV-2. This highlights the serious consequences that an HCoV infection can cause. Accordingly, this also reveals the clinical burden of such infections and the need for further investigation of these pathogens. Pneumonia is listed as one of the main causes of hospital admission and subsequent complications leading to death and is listed as the most typical and critical manifestation of COVID-19 [46]. The World Health Organization reports that the complication of pneumonia in a lower percentage of patients with COVID-19 is moderate compared to patients infected with other respiratory pathogens [47]. Accordingly, there is a need to determine whether the clinical case burden of hospitalised patients with pneumonia and proven infection caused by endemic coronaviruses is similar to that of those infected with SARS-CoV-2 [48]. We observed a longer duration of hospital treatment in patients co-infected with HCoV compared with those mono-infected with SARS-CoV-2. Another study of ours reported a longer hospital stay in the remaining mixed infections of SARS-CoV-2 with other respiratory viruses, as along with a greater percentage of intensive treatments [23]. A similar trend has been reported in other studies [24]. Furthermore, participants who were co-infected with SARS-CoV-2 and eHCoV had approximately threefold higher CRP and lower saturation levels than those co-infected with other respiratory viruses. For each one-unit increase in CRP concentration, risk levels of severe events have been reported to increase by 5 % in patients with COVID-19 [49]. Elevated CRP levels are a typical clinical manifestation of severe COVID-19 [50], while normal or low levels of elevated CRP are seen early in infection caused by other respiratory viruses [51]. In this study, critical saturation levels of CRP were observed in patients with mixed infections with endemic coronaviruses and SARS-CoV-2, while those co-infected with other respiratory viruses had close to normal levels. Combinations of high CRP levels and low oxygen saturation levels indicate severe COVID-19 [49,52]. This explains the need for longer hospital treatment in such patients [25].

There is a considerable body of evidence on the bacterial causes of respiratory complications, often necessitating hospitalisation. However, data on HCoV and other respiratory viruses as causes of complications and hospitalisation is not sufficient [53]. Furthermore, the genetic structure of viruses suggests that they accumulate more mutations in a shorter time than bacterial pathogens [54]. This suggests the emergence of new viral pathogens with SARS-CoV-2-like pandemic potential. Expanding knowledge about the mechanism of transmission, symptoms, and complications that respiratory viruses cause, particularly endemic coronaviruses, would help to manage better and reduce the future consequences that a new pandemic would cause. The development of a wide range of diagnostic methods covering a wider range of respiratory pathogens will facilitate timely and accurate diagnosis and indirectly reduce the overuse of antibiotics.

Limitations of this study include the lack of clinical data on outpatients with HCoV infections, indicating that these infections were not covered. Another limitation of this study is the small number of confirmed co-infections between eHCoV and SARS-CoV-2, which is insufficient to make a definitive assessment of its clinical severity.

Despite the limitations, the strengths of this study stood out. Involving testing and clinical analysis of 17 respiratory viral infections and recognising that the symptoms of eHCoV infections among hospitalised patients are more similar to those of SARS-CoV-2 than to those of other respiratory viruses. Of particular significance was the identification of eHCoV co-infections with SARS-CoV-2 as a cause of severe COVID-19 in hospitalised patients.

Further research is needed to understand the impact of eHCoV infections. Future studies should focus on tracking the frequency and clinical manifestations of respiratory infection in mixed infections of eHCoV and other non-coronavirus respiratory viruses. This will help to understand the role of eHCoVs in various mixed infections and their relevance in aggravating the clinical condition of patients.

## 5 Conclusions

During the devastating COVID-19 pandemic, our study reported a low prevalence (1.7 %) of eHCoVs and a low rate (0.7 %) of HCoV co-infections in patients positive for SARS-CoV-2. Such mixed infections can lead to severe forms of COVID-19, requiring oxygen therapy and prolonged hospitalisation. Simultaneous screening for common respiratory pathogens using molecular methods is necessary to assess disease severity, prognosis, and timely administration of adequate treatment. The results of this study will support the development of control and prevention strategies in the post-pandemic period when SARS-CoV-2 circulates in parallel with the four types of HCoV as an endemic virus. Understanding the impact and clinical relevance of HCoV infections in future studies is important, specifically focusing on tracking the frequency and manifestations of respiratory infections in mixed infections with other non-coronavirus respiratory viruses.

## Funding

This article is supported by contracts KΠ-06-H 43/5/November 30, 2020/Molecular-genetic and clinical characteristics of human coronavirus and European Regional Development Fund through the Operational Program Science and Education for Smart Growth 2014–2020, Grant BG05M2OP001-1.002-0001-C04 Fundamental Translational and Clinical Investigations on Infections and Immunity. All opinions, conclusions, or recommendations expressed are those of the authors and do not represent the policy or position of the National Center of Infectious and Parasitic Diseases and the Medical University of Sofia. The funding agency had no role in the analysis and interpretation of the data, the writing of the manuscript, or the decision to submit the manuscript for publication.

## Data availability statement

This manuscript utilizes a database on the spread of respiratory viruses in Bulgaria, which is accessible at: https://grippe.gateway.bg/index.php.

## Transparency declaration

The corresponding author affirms that the manuscript is an honest, accurate, and transparent account of the study being reported; that no important aspects of the study have been omitted; and that any discrepancies from the study as planned have been explained. The authors have no conflicts of interest to declare. No funding was received for this work.

## CRediT authorship contribution statement

**I. Trifonova:** Writing – review & editing, Writing – original draft, Visualization, Validation, Supervision, Software, Resources, Project administration, Methodology, Formal analysis, Data curation, Conceptualization. **N. Korsun:** Validation, Resources, Data curation. **I. Madzharova:** Methodology, Formal analysis. **P. Velikov:** Validation, Software, Resources, Formal analysis. **I. Alexsiev:** Validation, Resources, Formal analysis. **L. Grigorova:** Methodology. **S. Voleva:** Software, Resources, Formal analysis. **R. Yordanova:** Software, Methodology, Formal analysis. **I. Ivanov:** Project administration. **T. Tcherveniakova:** Validation, Project administration. **I. Christova:** Validation, Investigation, Funding acquisition.

## Declaration of competing interest

The authors declare that they have no known competing financial interests or personal relationships that could have appeared to influence the work reported in this paper.
